# Improvements in French risk stratification score were correlated with reductions in mean pulmonary artery pressure in pulmonary arterial hypertension: a subanalysis of the Japan Pulmonary Hypertension Registry (JAPHR)

**DOI:** 10.1186/s12890-021-01398-6

**Published:** 2021-01-14

**Authors:** Yuichi Tamura, Hiraku Kumamaru, Kohtaro Abe, Toru Satoh, Hiroaki Miyata, Aiko Ogawa, Nobuhiro Tanabe, Masaru Hatano, Atsushi Yao, Ichizo Tsujino, Keiichi Fukuda, Hiroshi Kimura, Masataka Kuwana, Hiromi Matsubara, Koichiro Tatsumi, the Japan Pulmonary Hypertension Registry (JAPHR) Network

**Affiliations:** 1 Pulmonary Hypertension Center, International University of Health and Welfare Mita Hospital, Tokyo, Japan; 2 Department of Healthcare Quality Assessment, Graduate School of Medicine, The University of Tokyo, Tokyo, Japan; 3 Department of Cardiovascular Medicine, Kyushu University Hospital, Fukuoka, Japan; 4 Department of Cardiology, Kyorin University School of Medicine, Mitaka, Japan; 5 Department of Health Policy and Management, Keio University School of Medicine, Tokyo, Japan; 6 Department of Clinical Science, National Hospital Organization Okayama Medical Center, Okayama, Japan; 7 Pulmonary Hypertension Center, Chibaken Saiseikai Narashino Hospital, Narashino, Japan; 8 Department of Cardiovascular Medicine, The University of Tokyo Hospital, Tokyo, Japan; 9 Division for Health Service Promotion, The University of Tokyo, Tokyo, Japan; 10 First Department of Medicine, Hokkaido University Hospital, Sapporo, Japan; 11 Department of Cardiology, Keio University School of Medicine, Tokyo, Japan; 12 Department of Pulmonary Medicine, Nippon Medical School Graduate School of Medicine, Tokyo, Japan; 13 Department of Allergy and Rheumatology, Nippon Medical School Graduate School of Medicine, Tokyo, Japan; 14 Department of Respirology, Graduate School of Medicine, Chiba University, Chiba, Japan

**Keywords:** Pulmonary arterial hypertension, Registry, Risk stratification, Mean pulmonary arterial pressures

## Abstract

**Background:**

Since there was no previous report, we analyzed the relationship between French Risk Stratification parameters in pulmonary arterial hypertension (PAH) and mean pulmonary arterial pressures (mPAP) using Japan PH Registry (JAPHR) national-wide cohort.

**Methods:**

We enrolled 108 patients with PAH from JAPHR from previous reported cohort and analyzed the relations between French Risk Stratification scores and hemodynamic improvements.

**Results:**

The ratio meeting 0 to 4 French Risk Stratification score was 21.3%, 31.5%, 32.4%, 13.0%, and 1.9% at baseline, and 6.5%, 23.2%, 33.3%, 23.2%, 13.9% at follow-up, respectively. The improvements in the number of criteria met were associated both with mPAP at follow-up (*p* = 0.03) and with the improvements in mPAP (*p* < 0.001).

**Conclusion:**

The improvements in French Risk Stratification may become a marker of improved hemodynamics including mPAP.

## Background

In the ESC/ERS pulmonary hypertension (PH) guidelines, right atrial pressure (RAP) and cardiac index (CI) have been proposed as hemodynamic parameters for pulmonary arterial hypertension (PAH) risk stratification [1]. Before the advent of current pulmonary vasodilators, as PAH progresses and the right ventricle dilates and fails, the mean pulmonary arterial pressure (mPAP) declines, resulting in the poor survival of patients with PAH. Therefore, past guidelines for PAH treatment recommended normalization of right ventricular function as a treatment goal, which is defined as RAP < 8 mmHg and CI > 2.5 L/min/m^2^. As a result, mPAP has been excluded as not only as a therapeutic target but also as a risk stratification marker. Recently, the French registry group proposed the French Risk Stratification for PAH, which includes RAP and CI as risk stratification markers at the time of diagnosis and during treatment [2]. The reliability of these risk stratification parameters has been supported in multiple conditions and registries [3]. However, with the progress of therapeutic strategies, including combination therapy, recent clinical observations suggest the possibility that mPAP may be an independent treatment goal. Ogawa et al. [4] reported that the survival rate of patients with mPAP ≤ 46 mmHg at follow-up was significantly better than that of patients with mPAP > 46 mmHg in 141 patients with idiopathic/heritable PAH at 3 PH centers in Japan. Thus, we expected that the current treatment strategy of using combination therapy improves not only the CI but also mPAP during follow-up in patients with PAH.

In this study, we aimed to analyze the changes in French Risk Stratification parameters and mPAP from baseline to the follow-up period using the Japan PH Registry (JAPHR) nationwide cohort.

## Methods

Patients registered in the JAPHR before 2013 and who initiated PH-specific treatment in or before March 2013 were included in the study. We extracted patients’ clinical information on WHO FC / NYHA classification, 6-min walking distance (6MWD), RAP, CI, and mPAP at baseline and at the first follow-up visit which was already reported [5]. We then categorized patients based on the number of risk stratification criteria that each patient met: i.e., NYHA classification I/II, 6MWD > 440 m, RAP < 8 mmHg, and CI > 2.5 L/m^2^, both at baseline and at the first follow-up. We also categorized patients into 3 groups based on the change in the number of criteria met between baseline and follow-up: improved (number of factors increased), unchanged (number of factors unchanged) and worsened (number of factors reduced). We compared the distribution of mPAP across these different risk groups using a Kruskal–Wallis test. Tests were all two-sided and p-values < 0.05 were considered statistically significant. This study was approved by the ethics committee of International University of Health and Welfare (Approval #5-19-11), and followed the Declaration of Helsinki and the ethical standards of the responsible committee on human experimentation. All patients provided written informed consent to participate.

## Results

**Fig. 1:**
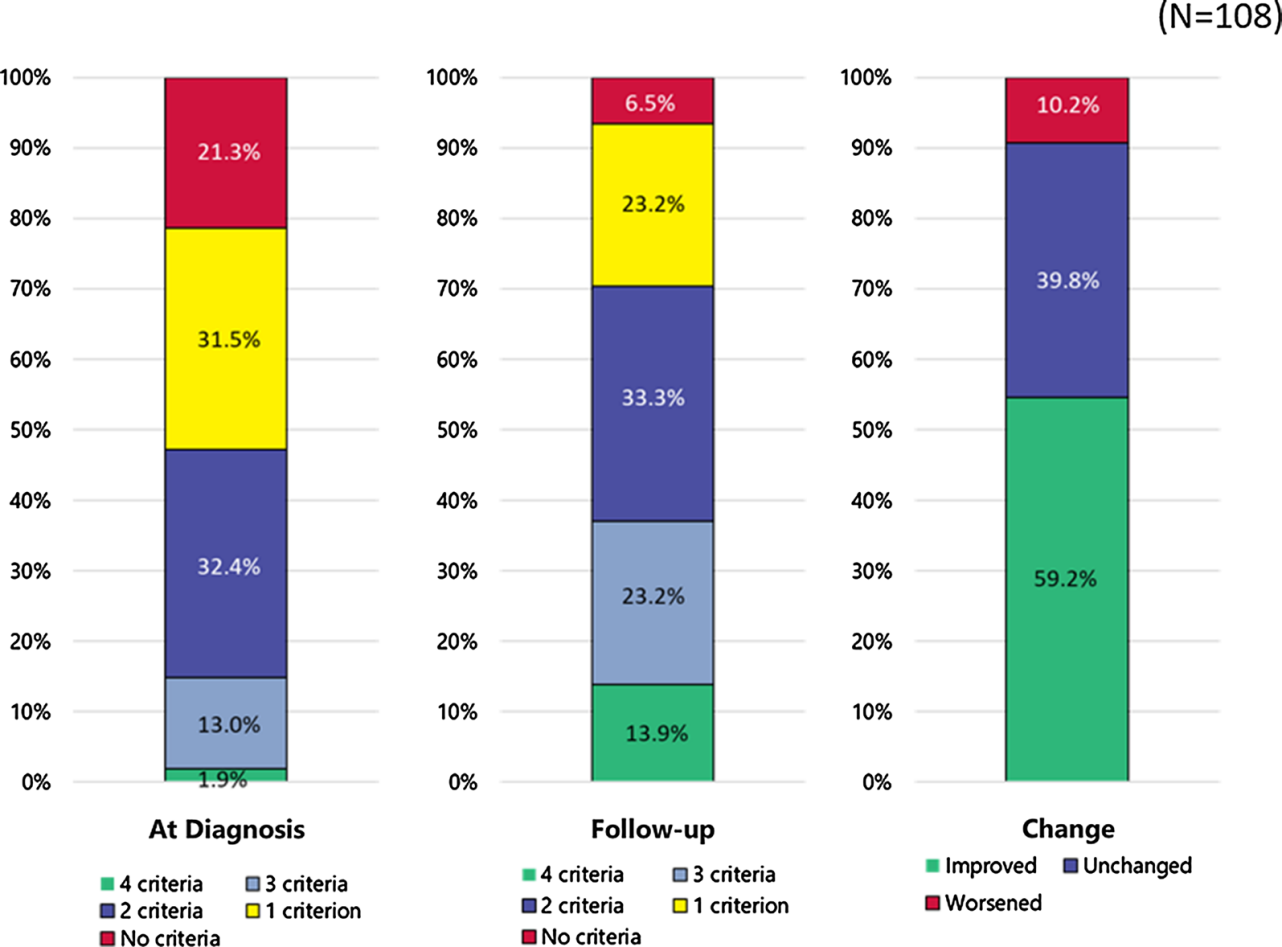
French risk stratification scores in the JAPHR cohort

**Fig. 2:**
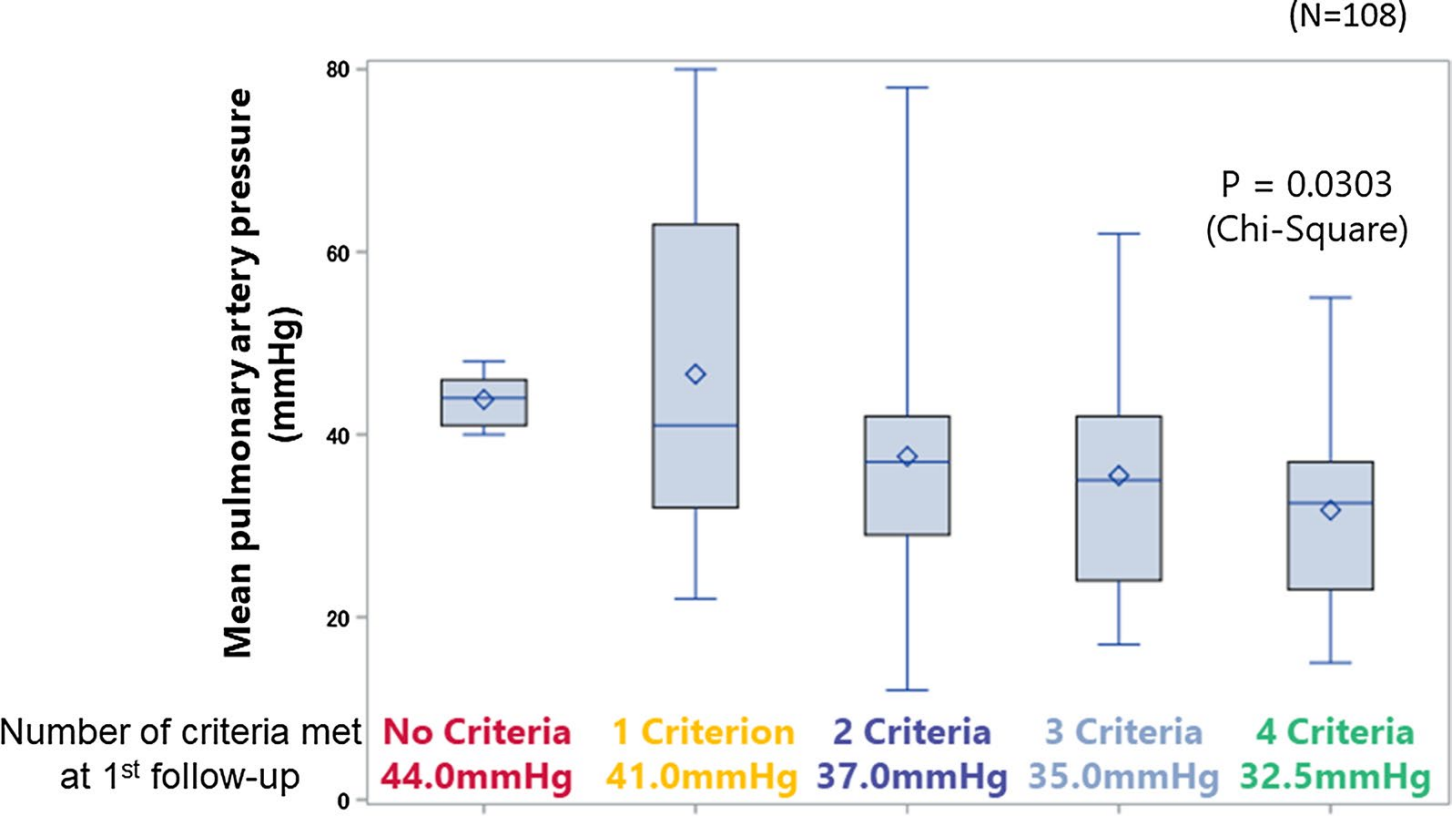
Correlation between French risk stratification score and hemodynamic improvements at the first follow-up

**Fig. 3:**
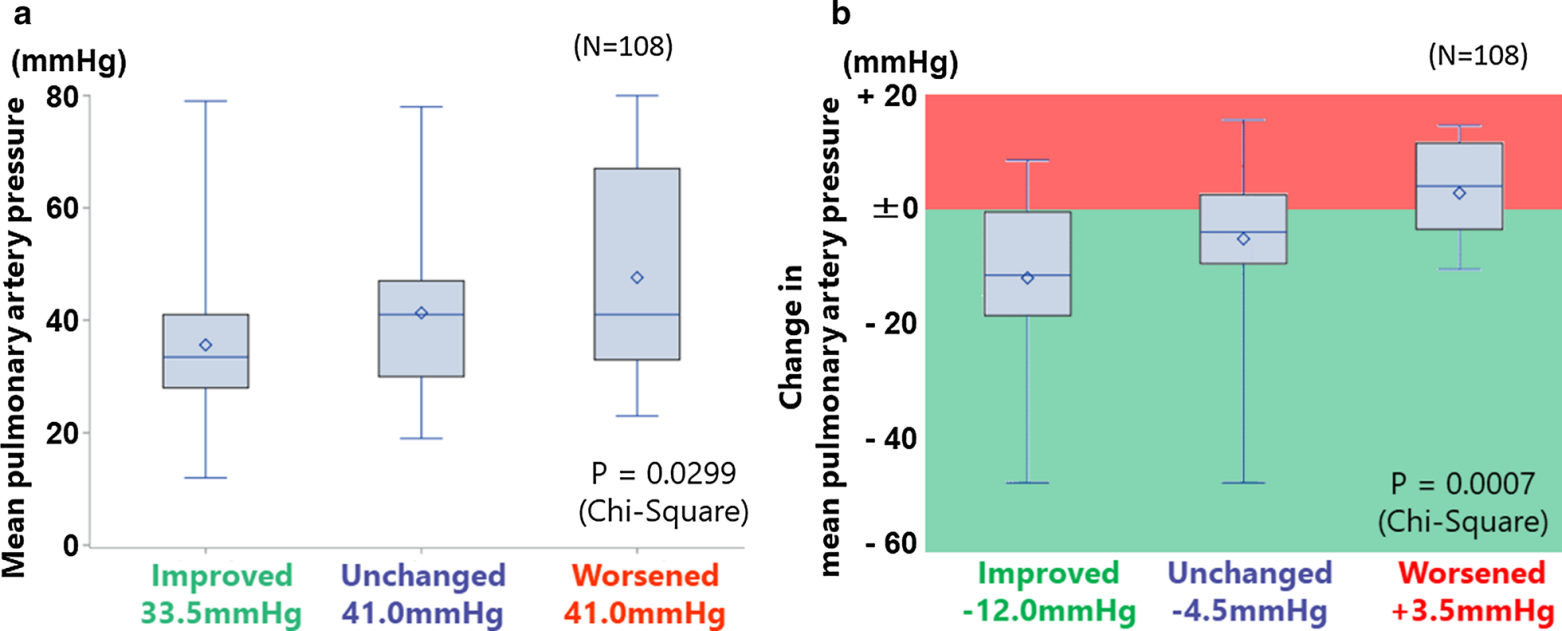
Relationship between the improvements in French risk stratification score and mean pulmonary artery pressure. **a** mean pulmonary artery pressures in the 3 groups of patients according to the improvement in French risk stratification score. **b** changes of mean pulmonary artery pressures on 3 groups of patients according to the improvement in French risk stratification score

We identified 108 patients matching the inclusion criteria in the database. The number of patients meeting each of the risk stratification criterion at baseline were 37 (34.3%), 10 (9%), 72 (67%), and 35 (33%) for NYHA I/II, 6MWD > 440 m, RAP < 8 mmHg, and CI ≥ 2.5 L/min/m^2^, respectively. At the first follow-up, the corresponding values were 59 (54.6%), 25 (23.2%), 84 (77.8%), and 64 (59.3%). The number of patients meeting 0 to 4 criteria was 23 (21.3%), 34 (31.5%), 35 (32.4%), 14 (13.0%), and 2 (1.9%) at baseline, and 7 (6.5%), 25 (23.2%), 36 (33.3%), 25 (23.2%), 15 (13.9%) for 0 to 4 at follow-up, respectively (Fig. 1). The median mPAP at the first follow-up decreased with the increase in criteria met: 44.0 mmHg for 0, 41.0 mmHg for 1, 37.0 mmHg for 2, 35.0 mmHg for 3, and 32.5 mmHg for 4 (*p* = 0.03, Fig. 2). Categorizing the patients into groups based on the change in the number of criteria met revealed that 58 (53.7%) improved, 30 (27.8%) were unchanged, and 10 (9.3%) worsened (Fig. 1). This grouping was associated with both mPAP at follow-up (*p* = 0.03) and the improvement in the mPAP at follow-up from baseline (*p* < 0.001, Fig. 3).

## Discussion

The evaluation of French risk stratification at baseline and follow-up in the JAPHR cohort revealed that the risk stratification score correlated with mPAP at follow-up. It was also found that improvements in the risk stratification score correlated with those in mPAP.

The most important point in this study is that improvement in the risk stratification score and hemodynamic parameters are closely related, especially in mPAP, in the JAPHR cohort. NIH registry data [6] indicated that not only increased RAP and decreased CI but also increased mPAP were associated with increased mortality in patients with primary PH. However, until now, most randomized controlled trials found mPAP improvement to be a poor prognosis factor [7]. One possible reason is that multiple monotherapy studies only showed minor improvements in mPAP, meaning that monotherapy itself has less capacity to improve mPAP. However, a report of triple combination therapy suggests that combination therapy including epoprostenol may improve not only the CI but also mPAP [8]. In addition, a JAPHR study reported that 85% of patients on upfront combination therapy had a ≥ 20% reduction in mPAP at the follow-up period, resulting in beneficial outcomes [5]. Therefore, in treatment with combination therapy, it is expected that lowering the risk stratification score will lead to a hemodynamic improvement in pulmonary circulation associated with not only right ventricular function but also mPAP. Regarding a novel treatment algorithm targeting risk stratification proposed in Nice 2018 [3], the results of the present study suggest that this algorithm improves not only risk stratification scores but also mPAP.

This study has some limitations. First, this cohort has data from advanced PH centers in Japan; as previously reported, the mortality and lung transplant events were less frequent and the prognosis was better than those of existing reports [5]. Therefore, it was difficult to verify any correlations between risk stratification score and prognosis at the initial visit or follow-up, as reported previously in other studies. However, absolute improvements in hemodynamics, as well as in right heart failure and symptoms contained in the risk stratification score, may indicate that patients in this cohort had a better prognosis. Second, a decrease in mPAP sometimes originate from worsening heart failure or decreases in cardiac output. However, since the risk stratification score includes indices such as CI and RAP, it is not expected that the decrease in mPAP was due to the progression in cardiac dysfunction. Moreover, only rare cases suffered from decreased mPAP associated with cardiac output impairment in our cohort because this cohort had better prognosis concomitant with hemodynamic improvements [5]. Third, this study was conducted in the cohort up to 2013, and the number of cases was small compared to the original French report [2]. However, compared with the French cohort, the distribution of patients in each risk score after the initial treatment did not differ, which validates the risk score in Japanese patients. Therefore, this study may contribute to the future universalization of evaluation methods for therapeutic interventions.

## Conclusion

In conclusion, the assessment of risk stratification score improvements, may become a novel marker of improved hemodynamics, including mPAP, in JAPHR.

## Abbreviations

PH: Pulmonary hypertension
RAP: Right atrial pressure
CI: Cardiac index
PAH: Pulmonary arterial hypertension
mPAP: Mean pulmonary arterial pressure
JAPHR: Japan pulmonary hypertension registry
WHO FC: World Health Organization functional classification
NYHA: New York Heart Association
6MWD: 6-Min walking distance
NIH: National Institutes of Health

## Availability of data and materials

All clinical data were obtained from medical records in each active centers. 11The data that support the findings of this study are available from Japan PH Registry (nonprofit organization) but restrictions apply to the availability of these data, which were used under license for the current study, and so are not publicly available. Data are however available from the authors upon reasonable request and with permission of Japan PH Registry.
